# Curcumin alleviates experimental colitis in mice by suppressing necroptosis of intestinal epithelial cells

**DOI:** 10.3389/fphar.2023.1170637

**Published:** 2023-04-07

**Authors:** Yuting Zhong, Ye Tu, Qingshan Ma, Linlin Chen, Wenzhao Zhang, Xin Lu, Shuo Yang, Zhibin Wang, Lichao Zhang

**Affiliations:** ^1^ Department of Pharmacy, Shanghai Municipal Hospital of Traditional Chinese Medicine, Shanghai University of Traditional Chinese Medicine, Shanghai, China; ^2^ Department of Pharmacy, Shanghai East Hospital, School of Medicine, Tongji University, Shanghai, China; ^3^ Department of Critical Care Medicine, School of Anesthesiology, Naval Medical University, Shanghai, China; ^4^ Longhua Hospital Affiliated to Shanghai University of Traditional Chinese Medicine, Shanghai, China

**Keywords:** curcumin, necroptosis, colitis, intestinal epithelial cells, RIP3

## Abstract

Curcumin, the primary bioactive substance in turmeric, exhibits potential therapeutic effects on ulcerative colitis. However, its mechanism for regulating necroptosis in colitis has not been fully elucidated. In this study, the effect of curcumin on experimental colitis-induced necroptosis of intestinal epithelial cells was investigated, and its molecular mechanism was further explored. We found that curcumin blocked necroptosis in a dose-dependent manner by inhibiting the phosphorylation of RIP3 and MLKL instead of RIP1 in HT-29 cells. Co-Immunoprecipitation assay showed that curcumin weakened the interaction between RIP1 and RIP3, possibly due to the direct binding of curcumin to RIP3 as suggested by drug affinity responsive target stability analysis. In a classical *in vivo* model of TNF-α and pan-caspase inhibitor-induced necroptosis in C57BL/6 mice, curcumin potently inhibited systemic inflammatory responses initiated by the necroptosis signaling pathway. Then, using a dextran sodium sulfate-induced colitis model in C57BL/6 mice, we found that curcumin inhibited the expression of p-RIP3 in the intestinal epithelium, reduced intestinal epithelial cells loss, improved the function of the intestinal tight junction barrier, and reduced local intestinal inflammation. Collectively, our findings suggest that curcumin is a potent targeted RIP3 inhibitor with anti-necroptotic and anti-inflammatory effects, maintains intestinal barrier function, and effectively alleviates colitis injury.

## 1 Introduction

The incidence of inflammatory bowel disease (IBD) has shown an upward trend year by year, but the etiology and pathogenesis of IBD are still not fully understood. Ulcerative colitis (UC), as a typical IBD, is characterized by chronic and recurrent inflammation of the gastrointestinal tract and has become a refractory gastrointestinal disease due to the lack of effective treatment (Katz, 2002). Intestinal epithelial cells (IECs) play vital roles in host defense, maintenance of mucosal homeostasis, and immune responses (Eissa et al., 2019), among which apoptosis of IECs is an important early event in the progress of UC (Iwamoto et al., 1996). Most previous studies have reported that excessive IECs apoptosis, regulated by multiple signaling pathways, disrupts the epithelial barrier function, contributing to chronic inflammatory bowel diseases (Schulzke et al., 2009; Seidelin and Nielsen, 2009).

In addition to apoptosis, there may be many other forms of cell death involved in UC. Of note is necroptosis, a caspase-independent programmed cell death, in which receptor-interacting serine/threonine protein kinases 1 and 3 (RIP1/3) play an important role (Cho et al., 2009; Zhang et al., 2009). It has been confirmed that altered intestinal epithelial necroptosis contributes to uncontrolled microbial translocation and amplifying inflammation (Vereecke et al., 2011; Günther et al., 2013; Pasparakis and Vandenabeele, 2015). The occurrence of necroptosis is positively correlated with the degree of intestinal injury. Inhibition of necroptosis by either chemical or genetic intervention can reduce intestinal injury and is considered a potential therapeutic strategy for the treatment of IBD.

One potential approach to treating IBD is oral natural products that precisely target inflamed areas of the colon to reduce unwanted side effects and improve therapeutic efficacy. Curcumin, a yellow natural product isolated from the roots of the turmeric plant, displays multiple pharmacological properties, including anti-cancer, anti-oxidant, anti-bacterial, anti-inflammatory, and neuroprotective (Bandgar et al., 2012; Dai et al., 2013). Several preclinical studies have demonstrated that curcumin effectively protects the intestinal mucosa and repairs intestinal tissue function (Holt et al., 2005; Yue et al., 2019). Clinical studies further reveal that curcumin combined with conventional drugs can effectively maintain UC remission and prevent recurrence (Simadibrata et al., 2017; Sadeghi et al., 2020). In the dextran sulfate sodium (DSS)-induced colitis model, curcumin is also found to repair intestinal mucosa and significantly ameliorate intestinal inflammation (Camacho-Barquero et al., 2007; Villegas et al., 2011).

Recently the effects of curcumin on necroptosis have garnered significant interest. Li *et al.* reported that curcumin has a protective effect on aflatoxin B1-induced liver necroptosis and inflammation (Li et al., 2022). Dai *et al.* found that curcumin protects primary cortical neurons from iron-induced neurotoxicity by attenuating necroptosis (Dai et al., 2013). Nevertheless, the regulatory effect of curcumin on colitis-associated intestinal epithelial necroptosis has yet to be reported. Therefore, the aim of this study is to investigate whether the protection of curcumin in DSS-induced colitis correlates with its inhibition of intestinal epithelial necroptosis.

## 2 Materials and methods

### 2.1 Reagents and primers

Curcumin, Demethoxycurcumin, Bisdemethoxycurcumin, SM-164 Hydrochloride, Necrostain-1, and Cycloheximide were purchased from MedChemExpress; z-VAD-fmk was purchased from TargetMol; mouse-TNF-alpha and human-TNF-alpha were purchased from Novoprotein. Anti-RIP1(3493S), anti-phospho-RIP1 (65746s, human-specific), anti-phospho-RIP1(38662S, mouse-specific) were purchased from Cell Signaling Technology. Occludin (33–1,500) and ZO-1 (61–7,300) antibodies were purchased from Invitrogen, anti-GAPDH (ab181602), anti-RIP3/p-RIP3 (ab209384, human-specific), anti-MLKL (ab184718, human-specific), anti-phospho-MLKL (ab187091, human-specific), anti-MLKL (66675-1-Iq, mouse-specific), anti-phospho-MLKL (ab196436, mouse-specific), anti-RIP3(17563-1-AP, mouse-specific), anti-phospho-RIP3 (ab195117, mouse-specific) were purchased from Abcam. IRDye 800CW goat anti-mouse secondary antibody (926–32210) and 680RD goat anti-rabbit secondary antibody (926–68071) were obtained from LI-COR Biosciences.

### 2.2 *In vivo* experiment

#### 2.2.1 Animal

Four weeks of C57BL/6 mice were purchased from SIPPR-BK biochemistry Co. (Shanghai, China, SCXK 2018-0006). In a plastic cage (6 mice/cage), standard laboratory food and water were freely provided during the acclimation period and throughout the experiment. Animal experiments were approved by the Animal Care Committee of Shanghai Municipal Hospital of Traditional Chinese Medicine (2019SHL-KYYS-07).

#### 2.2.2 Ulcerative colitis model induced by DSS

Male C57BL/6 mice (20–23 g) at 6 weeks were randomly divided into five groups, including control group (CON), DSS group (DSS), curcumin 50 mg/kg group (CUR50), curcumin 100 mg/kg group (CUR100) and Necrostain 1 group (Nec-1), with 8 mice in each group. All mice were given 2% DSS (MP Biomedicals, 36,000–50,000 kDa) to induce UC for 10 days except the CON, which was given a normal diet and water. On the 11th day, curcumin group mice were intragastric administrated with 50 mg/kg and 100 mg/kg curcumin, respectively. Mice in the Nec-1 group were intraperitoneally injected with 5 mg/kg Necrostain-1 once a day, the DSS group was only given 95%CMC-Na+5% DMSO. The body weight, feces, and activity of mice were documented every day to calculate the disease activity index (DAI). On the 21st day, all mice were sacrificed by the cervical dislocation method, blood and colon tissue were collected.

#### 2.2.3 Systemic inflammatory response syndrome (SIRS)

C57BL/6 mice (6 weeks, 21–24 g) were randomly divided into Vehicle group (Vehicle), 50 mg/kg curcumin group (CUR50), 100 mg/kg curcumin group (CUR100) and Necrostain 1 group (Nec-1), with 12 mice in each group. 15 min before caudal vein injection of m-TNF-α, mice in each group were intraperitoneally injected with corresponding concentrations of curcumin and Necrostain-1, respectively. The Vehicle group was given the same dose of control solvent, and initial body temperature was recorded. 15 min later, the first dose of z-VAD-fmk (180 μg) was intraperitoneally injected. One hour after m-TNF-α was injected, the second dose of z-VAD-fmk (70 μg) was given.

### 2.3 Cell culture

Human HT-29 cells (NCI-DTP Cat#HT-29), L929 cells (ECACC Cat# 141 12101), or EOL-1 cells (DSMZ Cat# ACC-386) were cultured in a high-glucose DMEM (Procell) containing 10% FBS (Biological Industries,04-001-1ACS), 100 U/mL streptomycin/penicillin, and 1% glutamine, Throughout the experiment, cells with no more than 10 generations were grown in an incubator with 5% CO_2_ at 37°C.

### 2.4 *Induction of necroptosis,* apoptosis, *and cell viability assay*

HT-29 cells cultured in 96-well plates, each well containing 2 × 10^4^ cells. Curcumin and Necrostain-1 were added to the corresponding concentration. SM-164 hydrochloride (10 nM) and caspase inhibitor z-VAD-fmk (20 μM) were joined together half an hour before the h-TNF-α (2 ng/100 μL), a total of 12 h of stimulation in the incubator. EOL-1 cells cultured in 96-well plates, each well was seeded with 2 × 10^5^ cells. SM-164 hydrochloride (10 nM) and caspase inhibitor z-VAD-fmk (20 μM) were mixed with the corresponding concentrations of curcumin, and half an hour later h-TNF-α (2 ng/100 μL) was added for 6 h of co-stimulation. L929 cells were seeded in 96-well plates at 2 × 10^5^ cells per well, and after overnight growth, caspase inhibitor z-VAD-fmk (20 μM) was mixed with the corresponding concentration of curcumin using medium, and after half an hour h-TNF-α (2 ng/100 μL) was added for co-stimulation for 6 h. TCZ experiment is the use of cycloheximide (10 nM) and caspase inhibitor z-VAD-fmk (20 μM) and h-TNF-α (2 ng/100 μL) induced by 12 h. Apoptosis was induced by TS (h-TNF-α plus smac mimetic) or TC (h-TNF-α plus cycloheximide) stimulation for 24 h respectively. Cells were stimulated with a specific time, 100 μL CellTiter-LumiTM luminescent reagents (Beyotime, C0065L) were added to each well, 37°C incubation after 10 min, every well liquid was transferred to 96-well board, using SpectraMaxM5 microplate reader to measure luminescent value.

### 2.5 Western blot

2 × 10^4^ human colon cancer HT-29 cells grew to 90% in 6-well plates. Curcumin was pre-administered with different concentrations and then stimulated by TSZ (h-TNF-α + SM-164 + Z-VAD-FMK) to observe the anti-necrosis activity of curcumin. In another experiment, TSZ was used to stimulate for 2 h, 4 h, and 6 h respectively to observe the anti-necrosis effect of curcumin at different time points. The cells were lysed with NP40 (Beyotime, P0013F) to obtain supernatant. After the concentration was determined by BCA, Western blot was performed to detect the phosphorylated and total protein expression of RIP1, RIP3 and MLKL. The 20 μg sample was isolated by 10% SDS-PAGE and transferred to NC membrane, sealed with 5% Not-fat milk for 1 h, and then added with 5% Not-fat milk diluted primary antibody (1:1,000) overnight at 4°C. On the second day, a fluorescent secondary antibody (1:8,000) was incubated in the dark for 1 h. Western blots were displayed and analyzed by Image Studio Ver 5. 2 system.

### 2.6 Immunoprecipitation

Human colon cancer HT-29 cells stimulated by TSZ were lysed with NP40 to obtain supernatant. BCA Protein Assay Kit (Beyotime, P0010) was used to determine protein concentration in cell lysate. PBS was added to keep the concentration consistent, and RIP1 primary antibody was joined and incubated at 4°C in a shaker for 24 h. Magnetic beads add rec-ProteinG-Sepharose (Thermo Fisher Scientific, 101242) 4°C for 24 h incubation, add 2x loading acquired immune coprecipitation protein buffer, 100°C, and degeneration, using Western blot detection RIP1 with the combination of RIP3.

### 2.7 RNA extraction and quantitative real-time PCR

Colonic tissue RNA was obtained using RNA isoPlus reagent (TaKaRa, 9109) PrimeScript RT Master mixing kit (TaKaRa, RR036A). Power qPCR SYBR Green master mixture (Thermo Fisher Scientific, A25742) was performed for real-time fluorescence quantitative PCR on an instrument (Roche, LightCyccler96). The 2^−ΔΔCt^ method was used to calculate the relative mRNA expression. Primer information is as follows: *IL-1β*, 5′-GCA​ACT​GTT​CCT​GAA​CTC​AAC​T-3′ and 5′-ATC​TTT​TGG​GGT​CCG​TCA​ACT-3′; *IL-6*, 5′-TAGTC CTTCCT AC CC C AATTTCC′ and 5′-TAG​TCC​TTC​CTA​CCC​CAA​TTT​CC-3′; *CCR2*, 5′-AT C CAC​GGC​ATA​CTA​TCA​ACA​TC′ and 5′-CAAGG CTCACCATC ATC GT A G′; *CCR6*, 5′-GTT CAA​CTT​TAA​CTG​TGG​GAT​G-3′ and 5′-GGTGT CT C ACCATC ATC GT A G′; *GAPDH*, 5′AGGCGAGGACTTTCT-3′ and 5′-GG GGTCG TTGATGGCAACA-3′.

### 2.8 Drug affinity responsive target stability assay

The supernatant protein solution was collected after the cells were lysed with NP40 solution. The protein solution was evenly divided into the drug administration group and the blank control group. Curcumin was added to the drug administration group, and the same amount of DMSO was joined to the blank control group. The samples were incubated for 1 h at room temperature, followed by the corresponding dose of pronase protease (Roche, 10165921001) incubated on ice for 20 min and heated with 5xloading buffer. Using Western blot methods to analyze the samples.

### 2.9 FITC-dextran assay

The mice fasted for 4 h, and then orally gavage 600 mg/kg FITC-dextran (Sigma, FD4, mol wet:3,000–5,000) 4 h before death. After blood collection, the supernatant was obtained by centrifugation and at 1:5 diluted with PBS. Finally, the serum FITC-dextran levels were detected by SpectraMax M5 fluorescence assay (Ex: 488 nm, Em: 525 nm).

### 2.10 Immunofluorescence

Animal tissue specimens and paraffin-embedded intestinal sections were divided into 1 μm sections. The epithelial monolayer paraffin was removed and immunostained. The tissue slides were incubated overnight with antibodies at 4°C, and after 4 rinses with PBS, the second antibody was incubated for 1 h away from light. Next, stain with DAPI (Beyotime, C1005) for 10 min at room temperature. After the cells were rinsed with PBS, an anti-fluorescence quencher was dropped (Beyotime, P0191-3). Representative results were photographed by confocal microscopy (C2+, Nikon).

### 2.11 HE and TUNEL staining

4% paraformaldehyde was performed to fix with colon tissue. After dehydration, the tissue was embedded with paraffin, then sliced into 4 mm sections and stained with hematoxylin and eosin (G1005) and TUNEL (GB1502). Before staining, xylene was used to remove paraffin from sections. Finally, a microscopic examination (NIKON ECLIPSE TI-SR, Japan) was performed, and the images were collected and analyzed.

### 2.12 Statistical analysis

Statistical analysis was performed by GraphPad Prism 8.0. An ANOVA and Tukey’s *post hoc* tests were performed to evaluate experiments involving multiple groups. P < 0.05 was set as statistically significant in all figures. All data were represented by mean ± SD. Statistical tests and the number of repetitions (n) were given in the figure legends. ImageJ 8.0 was used to calculate the gray value of each Western blot. The mechanism diagram is drawn using Figdraw software.

## 3 Results

### 3.1 Curcumin inhibits necroptosis in HT-29 cell line

**FIGURE 1:**
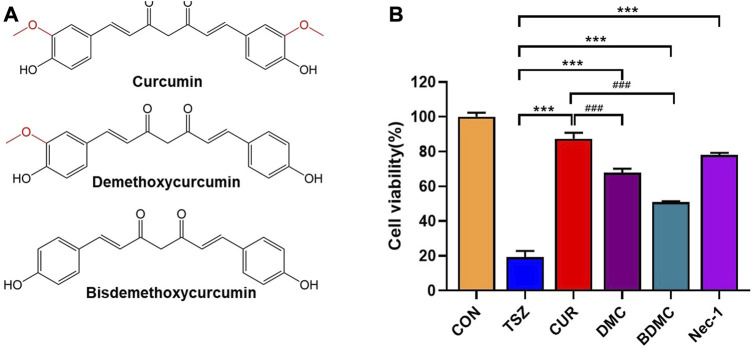
The anti-necroptotic activities of curcumin compounds were compared. **(A)** The chemical structural formula of curcumin, dimethoxycurcumin, and bisdemethoxycurcumin. **(B)**HT-29 cells were pretreated with curcumin, demethoxycurcumin, and bisdemethoxycurcumin, Necrostain-1 was used as a positive control, followed by a final concentration of 20 ng/ml h-TNF-α (T), 10 nM SM-164 Hydrochloride (S), and 20 mM z-VAD-fmk (Z) to induce necroptosis. (***p < 0.001, compared with TSZ group; ###*p* < 0.001, compared with CUR group).

The active ingredients of curcuminoid compounds extracted from turmeric mainly include curcumin (CUR), demethoxycurcumin (DMC), and bisdemethoxycurcumin (BDMC). Among them, curcumin (Dai et al., 2013; Lee et al., 2021; Chung et al., 2022; Li et al., 2022)and BDMC (Córdoba-David et al., 2020) have been reported to have anti-necroptosis activity. To further assess the anti-necroptosis activity of the three (Figure 1A), we first examined cell viability after pretreatment with these three curcuminoids compounds in TSZ (h-TNF-α + SM-164 + Z-VAD-FMK) induced necroptosis in human colon cancer HT-29 cells. The results showed that three curcuminoids compounds were both efficient in blocking TSZ-induced necroptosis in HT-29 cells (Figure 1B). Since curcumin exhibited the strongest anti-necroptotic activity, we further evaluated its biological effects in subsequent experiments. (Figure 1B).

**FIGURE 2:**
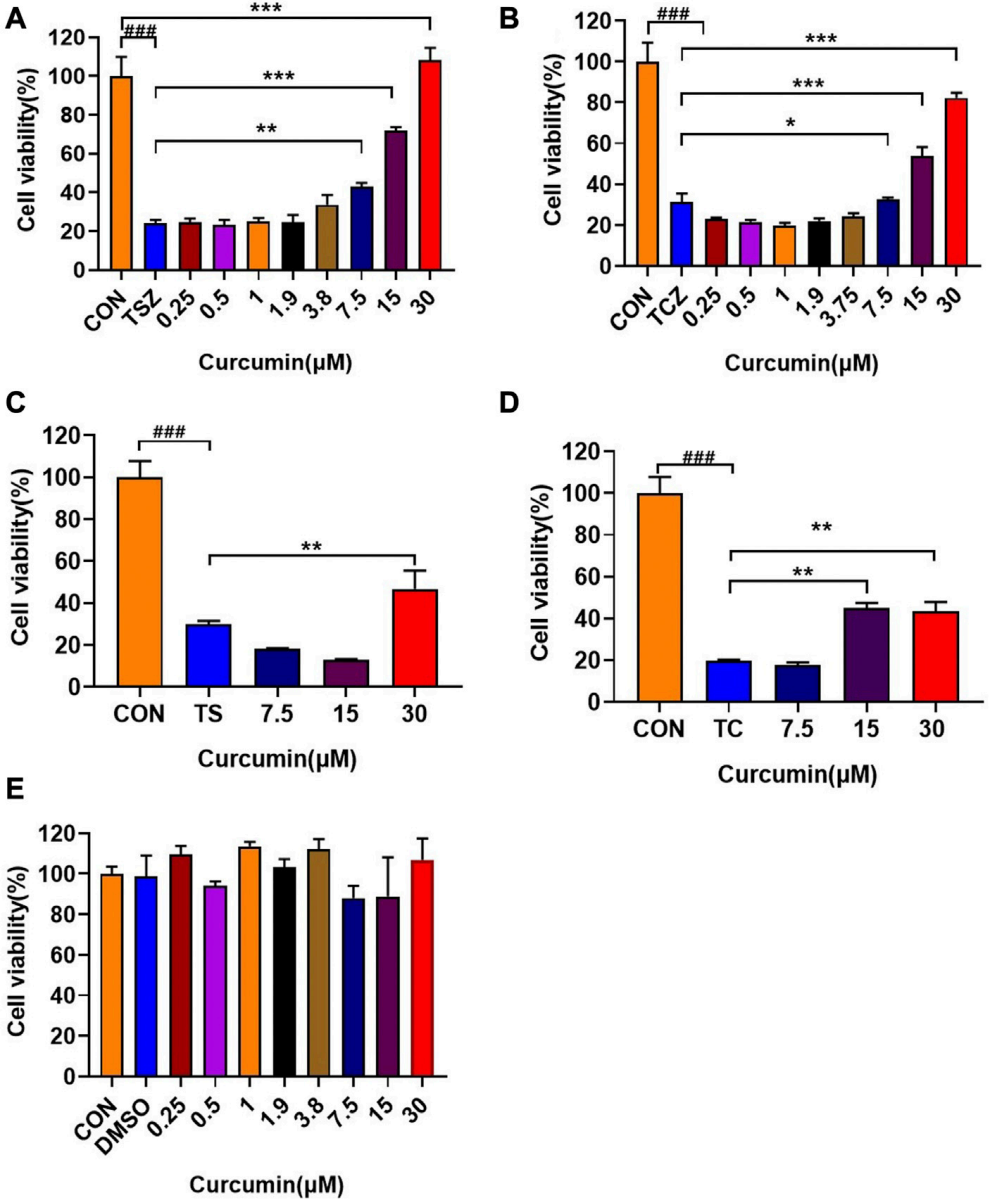
*In vitro* anti-necroptosis pharmacological activity of curcumin. **(A, B)** In TSZ (h-TNF-α + SM-164 + Z-VAD-FMK) and TCZ (h-TNF-α + cycloheximide + Z-VAD-FMK) assay, curcumin protected HT-29 cells in a dose-dependent manner. **(C, D)** Effects of curcumin on HT-29 cells in TS (h-TNF-α + smac mimetic) and TC (h-TNF-α + cycloheximide) induced apoptosis experiments. **(E)** Curcumin had no obvious toxic effect on HT-29 cells within 24 h (###*p* < 0.001, compared with CON group; *p < 0.05, **p < 0.01, ***p < 0.001 compared with the model group as indicated in the figure).

Curcumin could dose-dependently inhibit TSZ (Figure 2A) or TCZ (h-TNF-α + cycloheximide + Z-VAD-FMK) -induced necroptosis. The same anti-necroptosis results were demonstrated in EOL-1 (Supplementary Figure S1A, B) and L929 (Supplementary Figure S2A, B) cell lines, which have been identified as mature necroptotic cellular models (Figure 2B). Besides, we also found that curcumin showed weak protective effects at high concentrations in TS (h-TNF-α plus Smac mimetic) or TC (hT-NF-α plus cycloheximide) induced apoptosis model (Figures 2C,D). Moreover, we confirmed that curcumin alone had no cytotoxicity on HT-29 cells within 24 h at doses lower than 30 μM (Figure 2E). Collectively, these results indicated that curcumin effectively inhibited activation of necroptosis *in vitro*.

### 3.2 Curcumin inhibits necroptosis *in vitro* by selectively targeting RIP3

**FIGURE 3:**
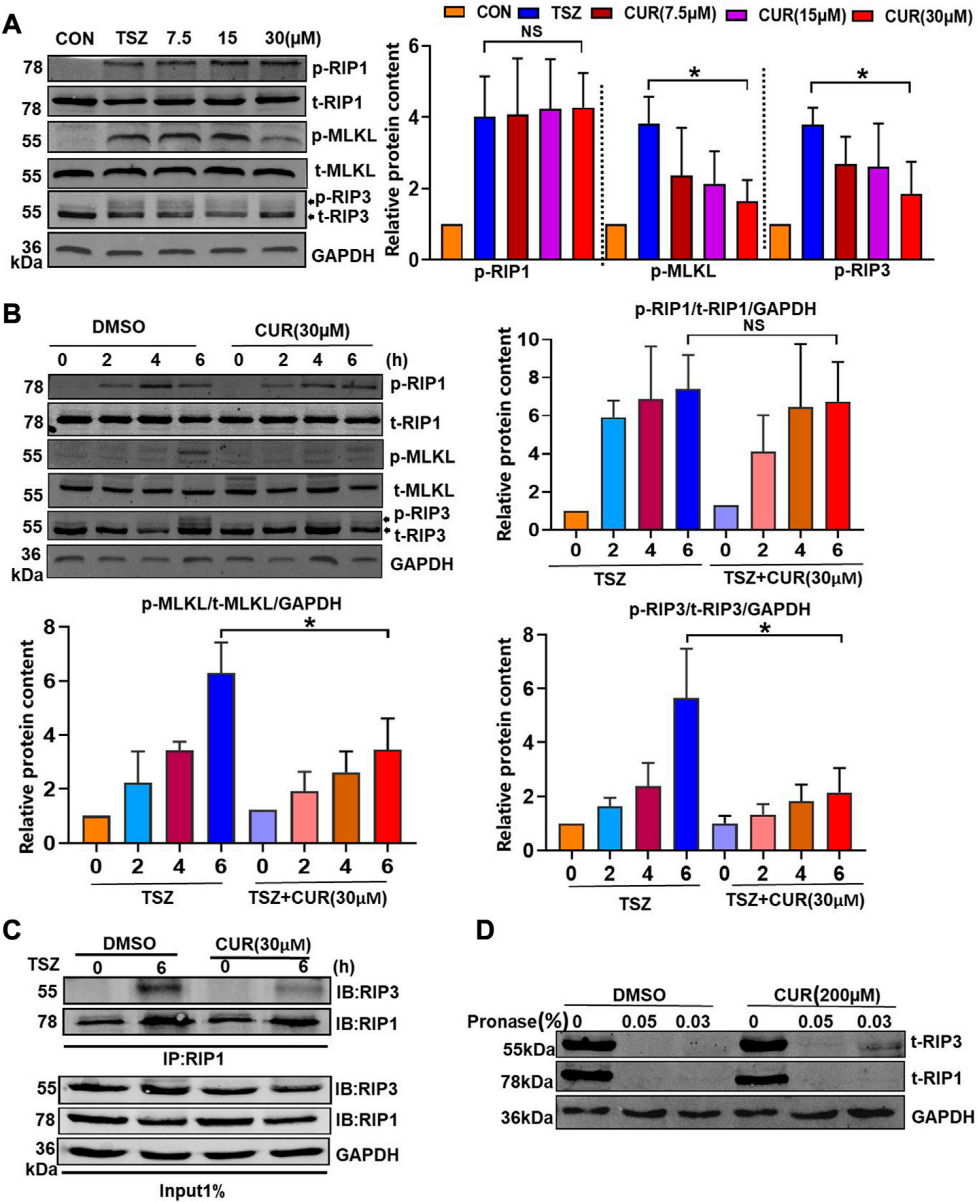
Molecular mechanism of curcumin blocking the activation of the necroptosis signaling pathway. **(A)** Curcumin inhibited p-RIP3 and p-MLKL in a dose-dependent manner. Statistical analysis of the gray value showed three independent repeated experiments. **(B)** The inhibitory effect of curcumin on p-RIP1, p-RIP3, and p-MLKL was observed using TSZ stimulation at different time points. **(C)** HT-29 cells were treated with DMSO or curcumin (30 µM) for 6 h. Cell lysates were immunoprecipitated with an anti-RIP1 antibody (IP: RIP1) and subjected to immunoblot analysis with the indicated antibodies. **(D)** Cell lysates from HT-29 were incubated with curcumin (200 µM) and DMSO for 1 hour, respectively, followed by the addition of 0.05% and 0.03% protease. The expression of RIP1 and RIP3 was then examined by immunoblotting. (*p < 0.05, compared with TSZ group).

The RIP1-RIP3-MLKL signaling pathway has been reported to be involved in the induction of necroptosis (Yang et al., 2016). Therefore, we examined the protein expression and phosphorylation of RIP1, RIP3, and MLKL after curcumin treatment in TSZ-induced HT-29 cells, aiming to elucidate the inhibitory effect of curcumin on the necroptosis pathway. Firstly, Western blotting showed that curcumin treatment dose-dependently downregulated the phosphorylation of RIP3 (p-RIP3) and phosphorylation of MLKL (p-MLKL), but had no influence on total protein expression of RIP3 (t-RIP3) and MLKL (t-MLKL) (Figure 3A). Interestingly, curcumin had no significant influence on the expression of p-RIP1 or t-RIP1 (Figure 3A). Furthermore, we found that curcumin could completely inhibit the p-RIP3 at 30 μM within 6 h. Subsequently, the downstream p-MLKL was also markedly inhibited (Figure 3B).

The formation of RIP1/3 complex bodies is necessary for the development of necroptosis (Cho et al., 2009). Therefore, we further evaluated the interaction between RIP1 and RIP3 by co-immunoprecipitation in curcumin-treated HT-29 cells. As compared with the DMSO-treated cells, the level of co-immunoprecipitated RIP3 decreased significantly in the curcumin-treated cells when RIP1 was immunoprecipitated with its antibody (Figure 3C). This suggested that curcumin could block TSZ-induced necrosome formation for inhibiting the necroptosis process. To examine whether curcumin directly interacted with RIP3, we performed drug affinity responsive target stability assay (DARTS) (Chen et al., 2019) to detect the potential interaction between curcumin and RIP3. In the sample of curcumin-treated HT-29 cells. It was found that RIP3 but not RIP1 was protected from protease digestion (Figure 3D), indicating that curcumin may prefer to interact with RIP3. Overall, these results suggested that curcumin may be a selective inhibitor of RIP3.

### 3.3 Curcumin inhibits systemic inflammatory response syndrome (SIRS) *in vivo*

**FIGURE 4:**
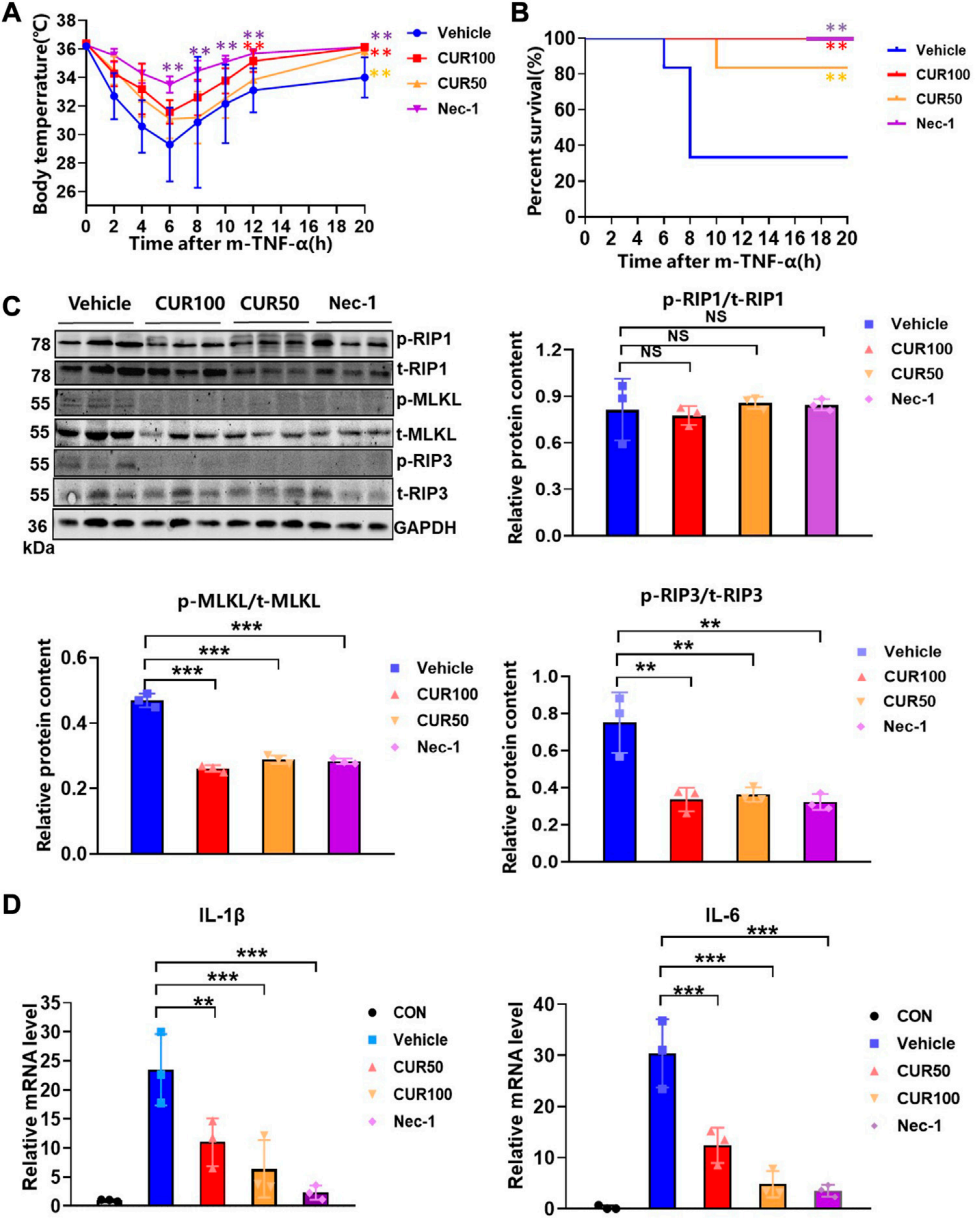
Curcumin potently inhibited systemic inflammatory responses initiated by the necroptosis signaling pathway in an *in vivo* model. **(A)** Body temperature of C57BL/6 mice (n = 6) injected with m-TNF-α (65 μg/kg) and z-VAD-fmk (250 µg) after treatment with indicated doses of curcumin (100 mg/kg) and the survival curves **(B)**. **(C)** The expression of necroptosis biomarkers in colon tissue was analyzed by Western blot. And quantitative analysis of gray scales of protein bands. **(D)** Quantitative analysis of inflammatory cytokine RNA in mouse colon tissue. (**p < 0.01, ***p < 0.001, compared with Vehicle Group).

To evaluate the anti-necroptosis activity of curcumin *in vivo*, we established a SIRS model characterized by hypothermia (Duprez et al., 2011). After intraperitoneal injection of z-VAD-fmk (180 μg) and curcumin (50 and 100 mg/kg) for 15 min, m-TNF-α (65 ug/kg) was injected *via* the tail vein into the mice. Half an hour later, a second dose of z-VAD-fmk (70 μg) was administered. The results showed that both Necrostatin-1 (Nec-1, RIP1-targeted inhibitor) and curcumin significantly protected mice from extremely low hypothermia within 20 h compared with the vehicle group (Figure 4A). Similarly, prophylactic curcumin greatly increased the survival of mice in a dose-dependent manner. Consistent with the Nec-1 group, the percent survival of mice in the CUR100 group was 100% and completely protected SIRS mice (Figure 4B). Moreover, curcumin displayed comparable levels of anti-necroptosis effects in female mice as it did in male mice (Supplementary Figure S3A, B).

We next sought to demonstrate whether curcumin prevents the occurrence of necroptosis by inhibiting RIP3 *in vivo*, we extracted mouse colon tissue proteins for immunoblotting, and the outcomes displayed that curcumin remarkably inhibited the expression of p-RIP3 and p-MLKL (Figure 4C). Moreover, the real-time quantitative PCR experiments showed that curcumin significantly reduced the mRNA levels of IL-1β and IL-6 in intestinal tissues (Figure 4D). These findings suggested that curcumin pretreatment alleviated symptoms, death rates, and the production of inflammatory cytokines in necroptosis-characteristic SIRS mice.

### 3.4 Curcumin alleviates DSS-induced ulcerative colitis *in vivo*

**FIGURE 5:**
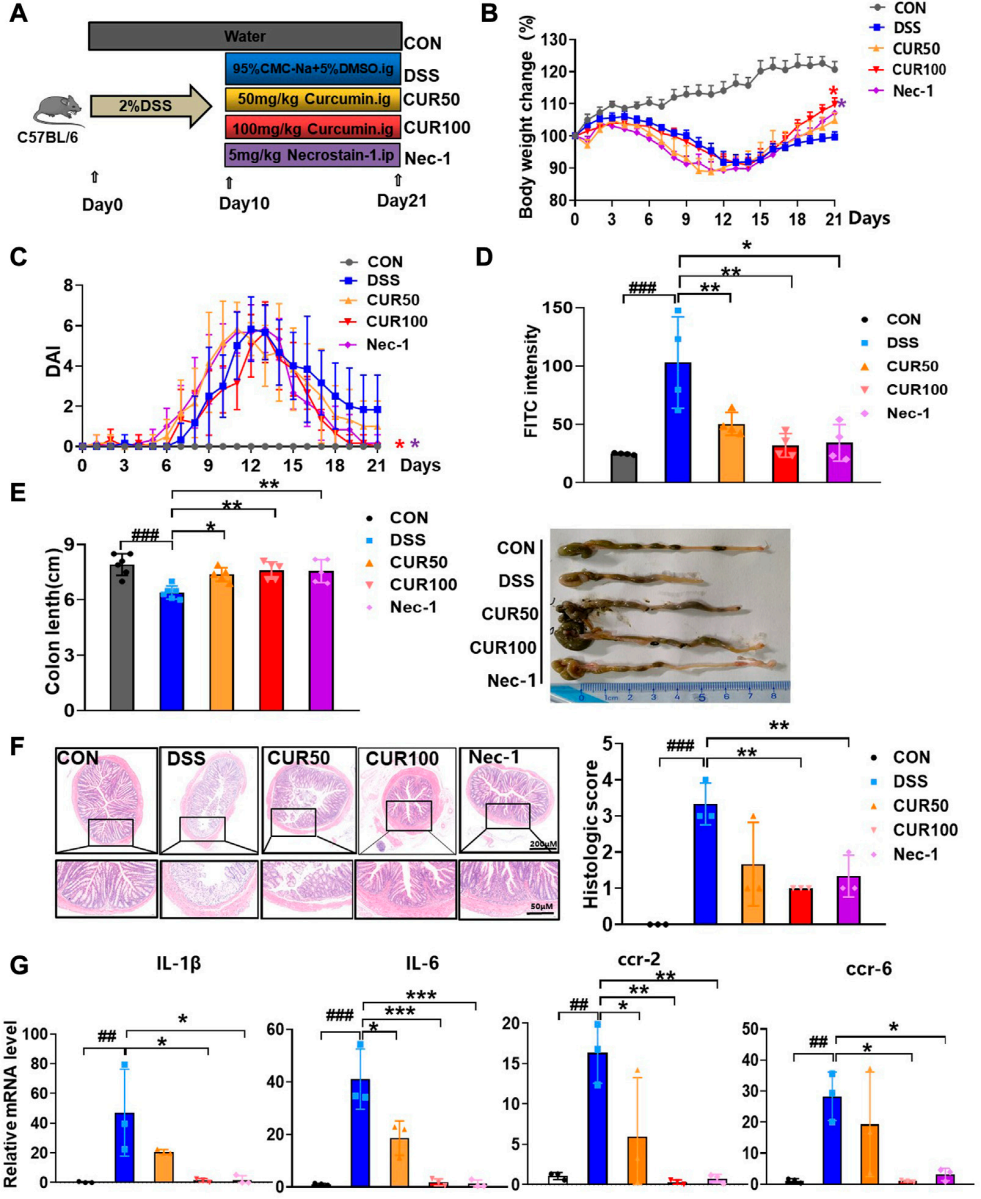
Curcumin alleviates symptoms of DSS-induced colitis. **(A)** Experimental protocol for the treatment of ulcerative colitis with curcumin (n = 6-8). **(B)** Body weight change. **(C)** Disease activity index (DAI). **(D)** FITC content of blood from mice 4 h after gavage. **(E)** Analysis of colon length and colon condition of mice in each group on the last day. **(F)** Representative images of H&E staining of mouse colon as well as histologic scores. **(G)** Quantitative analysis of inflammatory cytokine RNA in mouse colon tissue. (##*p* < 0.01, ###*p* < 0.001, compared with CON group; *p < 0.05, **p < 0.01, ***p < 0.001, compared with DSS group).

Curcumin has been reported to protect against DSS-induced UC (Holt et al., 2005; Camacho-Barquero et al., 2007), we speculated that curcumin may alleviate UC by inhibiting intestinal epithelial cell necroptosis. Therefore, we performed 2% DSS in C57BL/6 mice for 10 days to induce UC, followed by intragastric administration of curcumin to mice daily, intraperitoneal injection of Nec-1 as a positive control and evaluated the therapeutic efficacy of curcumin (Figure 5A). We observed that the trend of weight loss was curbed on the fourth day of treatment, and the body weight of the mice treated with curcumin was significantly restored as compared to that in the DSS group from the 11th day to the 21st day (Figure 5B). We measured clinical signs of DSS-induced UC in each group of mice by disease activity index (DAI). The index consists of three parameters (fecal consistency, rectal bleeding, and body weight changes of mice) and provides the order of severity of the inflammatory process associated with intestinal mucosal damage. As expected, the DAI of curcumin-treated mice was decreased compared to the DSS control group (Figure 5C).

In addition, as compared to the serum of DSS mice, the level of FITC-dextran in that of curcumin-treated mice was also robustly lower (Figure 5D), indicating that curcumin protected intestinal barrier function in DSS-induced colitis mice, with an effect comparable to that of Nec-1. We examined colon length after 10 days of curcumin treatment as it is a well-recognized indicator of DSS-induced colitis. As expected, administration of curcumin significantly prevented DSS-induced colon shortening, robustly ameliorated the ulcer area, reduced loss of mucosal epithelium and goblet cells, improved inflammatory infiltration, and overall decreased histopathological score (Figures 5E,F). Moreover, the mRNA expression of inflammatory cytokines (IL-6 and IL-1β) and chemokine receptors (Ccr-2 and Ccr-6) were significantly reduced through curcumin treatment (Figure 5G). Together, our results suggest that curcumin is effective in relieving DSS-induced colonic injury.

### 3.5 Curcumin ameliorates the loss of intestinal barrier function following DSS-induced colitis

**FIGURE 6:**
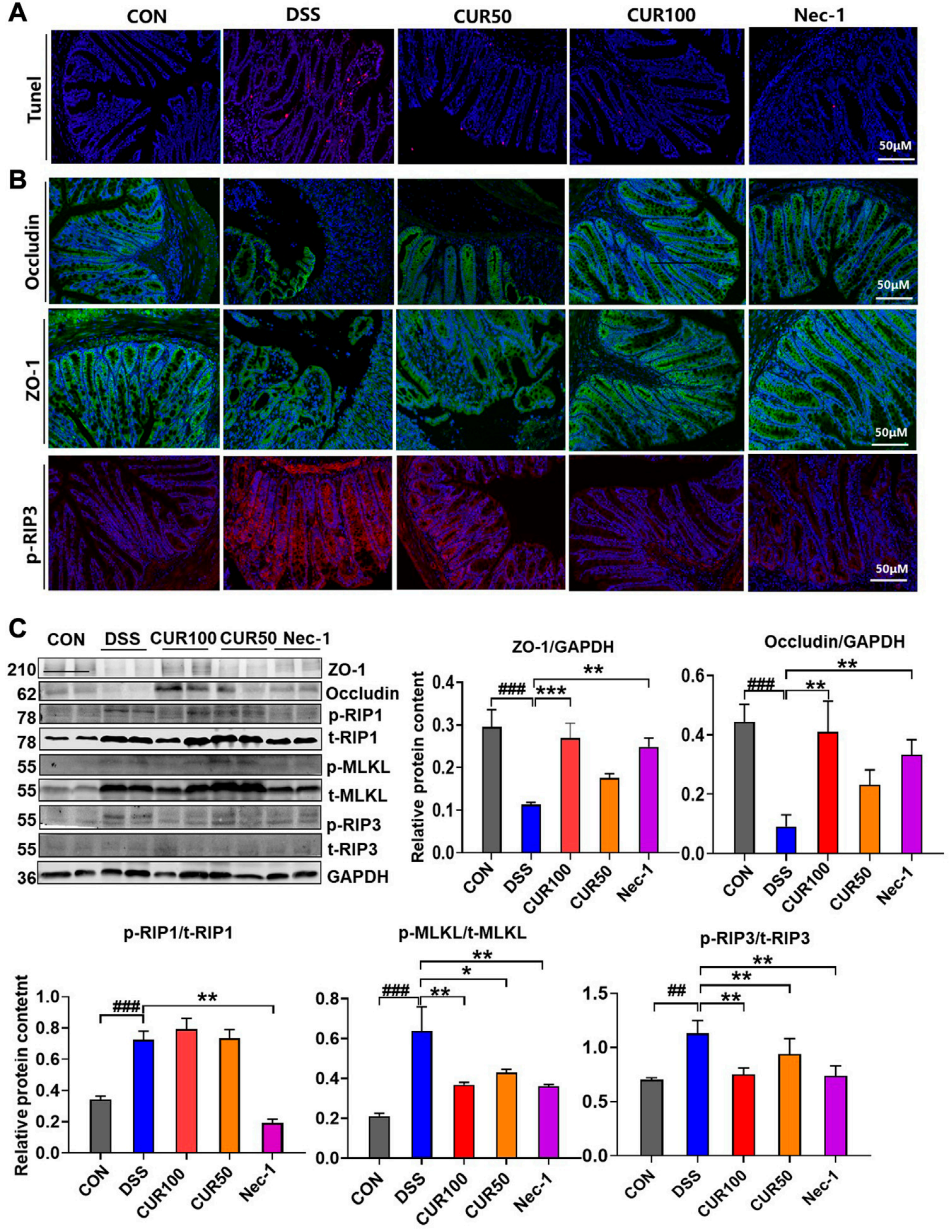
Curcumin inhibits intestinal epithelial necroptosis activation and tight junction barrier damage in mice with colitis. **(A)** Analysis of intestinal epithelial cell injuries by TUNEL. **(B)** Immunofluorescence staining showed intestinal epithelial TJ proteins including ZO-1 and occludin, and necroptosis-related protein p-RIP3. **(C)** The TJ proteins ZO-1 and occludin and necroptosis-related proteins p-RIP1, p-RIP3, and p-MLKL were detected by Western blot. And quantitative analysis of gray scales of protein bands. (##*p* < 0.01, ###*p* < 0.001, compared with CON group; *p < 0.05, **p < 0.01, ***p < 0.001, compared with DSS group).

It was demonstrated that administration of DSS resulted in intestinal epithelial barrier (IEB) dysfunction in mice, mainly manifested by increased paracellular permeability and decreased transepithelial electrical resistance (TER). In order to further examine the effect of curcumin on intestinal epithelial barrier function, we observed the death of intestinal epithelial cells in different groups of mice by TUNEL staining and found that curcumin can effectively reduce cell death in DSS-induced mice (Figure 6A). Subsequently, we performed occludin and Zonula occludens-1 (ZO-1) as tight junction (TJs) protein markers to test the IEB function of intestinal epithelial cells. The results of both immunofluorescence and Western blotting showed that curcumin treatment greatly raised the protein levels of occludin and ZO-1, but also decreased the expression of p-RIP3 and p-MLKL in the downstream pathway (Figures 6B,C). The above results indicated that curcumin could effectively inhibit the necroptosis pathway by reducing the p-RIP3 while enhancing the expression of occludin and ZO-1 in IECs to maintain intestinal epithelial barrier function.

## 4 Discussion

**FIGURE 7:**
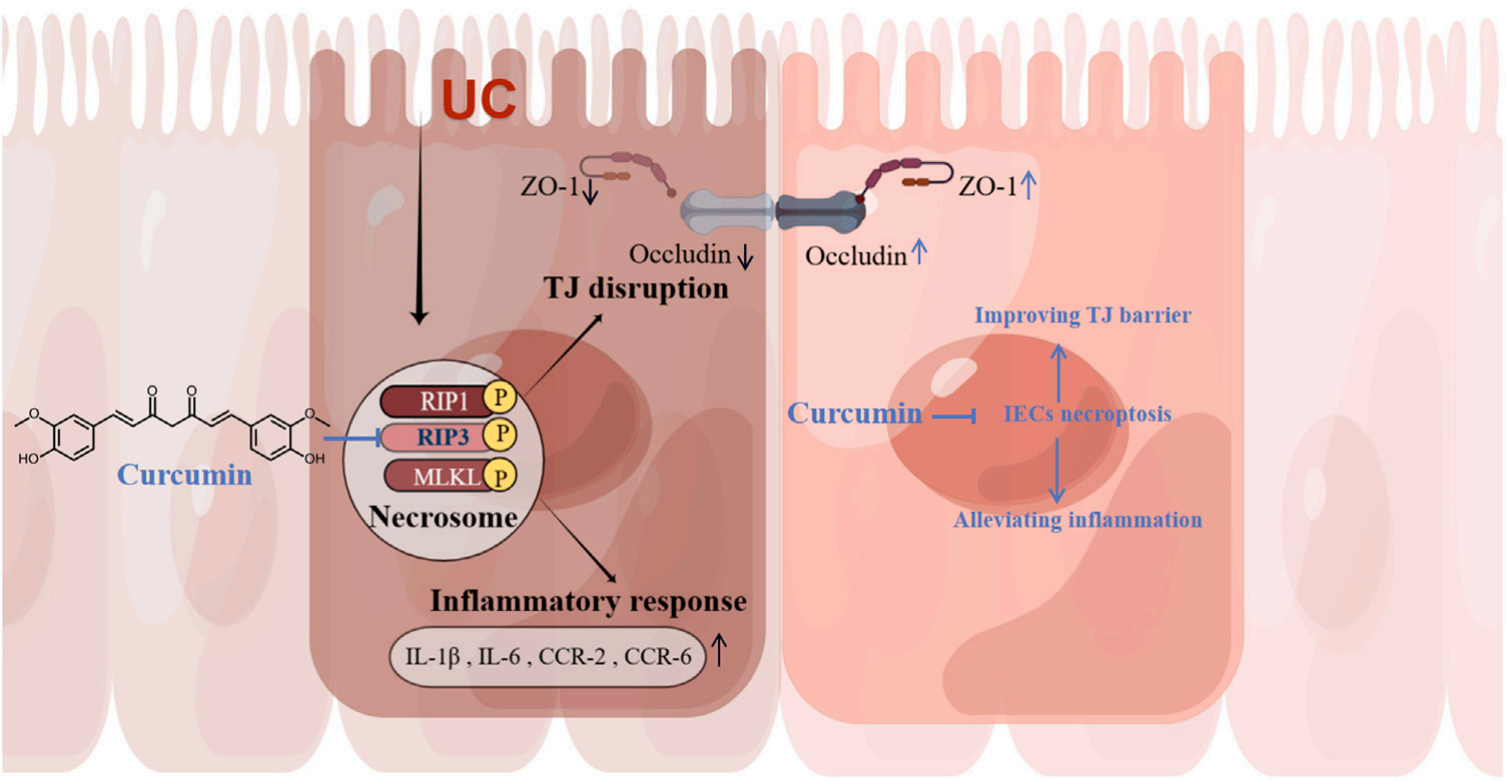
Curcumin alleviates experimental colitis in mice by suppressing necroptosis of intestinal epithelial cells. Curcumin inhibits IECs necroptosis by targeting RIP3 to ameliorate intestinal epithelial cell loss, local intestinal inflammation, and intestinal epithelial tight junction barrier injury.

In the present study, we found that the inhibitory effect of curcumin on necroptosis in intestinal epithelial cells may be associated with targeting RIP3. In an experimental colitis model, curcumin ameliorated necroptosis-associated intestinal epithelial cell loss, local intestinal inflammation, and intestinal barrier permeability damage associated with tight junction injury (Figure 7). These results suggest that curcumin may be a potential therapeutic agent for ulcerative colitis as an inhibitor of RIP3.

Turmeric has been used as an herbal remedy for various ailments for thousands of years. Curcuminoids are active ingredients extracted from the rhizome of Zingiberaceae (*Curcuma longa*) (Mazzio et al., 2016; Jeong, 2017; Sun et al., 2019), mainly including curcumin (71.5%), demethoxycurcumin (19.4%), and bisdemethoxycurcumin (9.1%), which are all diarylheptanoids (Pfeiffer et al., 2003; Hadi, 2018). The curcumin molecule contains multiple double bonds, as well as active groups such as phenolic hydroxyl and carbonyl, so it has strong physiological activity. Among them, curcumin is the most studied active ingredient, and it is also the major compound of curcuminoids to exert their medicinal values. The chemical properties of curcumin have an important influence on its biological activity. Several studies proposed the key role of methoxy groups and thiol-reactive α, ß unsaturated carbonyl groups of curcuminoids (Yang et al., 2017). We speculate that the biological ability of curcuminoids may be positively correlated with the number of methoxy groups. In our results, compared to DMC or BDMC, there were stronger inhibitory effects in curcumin treatment for TSZ-induced cell death, which implies that curcumin may have a greater value for the prevention and treatment of UC. However, the major obstacle to the clinical efficacy of curcumin is its poor bioavailability. Studies have shown that curcumin nanoformulations such as Polylactic-co-glycolic acid (PLGA) or hydroxypropyl beta-cyclodextrin (HP-β-CD) encapsulated curcumin significantly enhanced bioavailability after oral administration in rats (Shaikh et al., 2009). In addition, synthetic analogues can also enhance the solubility and stability of curcumin while preserving its pharmacological properties.

The potential medicinal values of curcumin have been widely recognized in terms of diseases with the discovery of its biological functions (Akbar et al., 2016). Moreover, it plays a vital role in protecting the intestinal mucosa and repairing intestinal epithelial barrier function (Holt et al., 2005; Shen and Durum, 2010). It has been reported that curcumin treatment effectively relieved experimental colitis by modulating the re-equilibration of Th17/Treg, closely related to the regulation of the IL-23/Th17 pathway (Wei et al., 2021). It was proved that curcumin could strongly alleviate DSS-induced experimental colitis by inhibiting NLRP3 inflammasome activation (Gong et al., 2018). Zhong et al. also stated that curcumin availably ameliorated DSS-induced UC through regulatory mechanism related to immune memory homeostasis of T-cells (Zhong et al., 2020). While the potential molecular modulatory effect of curcumin on necroptosis in DSS-induced UC is still unclear.

An insight into the mechanisms of necroptosis in UC may provide new therapeutic strategies for the prevention and treatment of IBD. Necrotizing apoptosis is a regulated form of programmed cell death independent of caspase, induced by cytokines, toll-like receptors, oxidative stress, or other death receptors (Dunai et al., 2011; Vanden Berghe et al., 2014; Humphries et al., 2015). Recent insights into the molecular mechanism of TNF-induced necroptosis (Vanlangenakker et al., 2011) reveal that RIP3, a critical factor for necroptosis, together with RIP1 and MLKL proteins, constitutes the main necroptosis signaling pathway (Sun et al., 2012; Zhang et al., 2016). When apoptosis is restrained, activated RIP1 binds to RlP3, generating a necrosome complex, followed by recruiting and phosphorylating MLKL. Then, the p-MLKL induces its oligomerization and translocation to the plasma membrane, causing necroptotic cell death by activating ion channels or promoting the formation of pore structures (Murphy et al., 2013; Dondelinger et al., 2014). Thus, necroptosis is generally considered to be a pro-inflammatory death on account of the release of intracellular immunostimulatory components following with cell lysis (Chan et al., 2015; Silke et al., 2015; Shlomovitz et al., 2017).

Recent evidence points to necroptosis involved in ischemic injury (Linkermann et al., 2012; Luedde et al., 2014), liver injury (Dara et al., 2016), systemic inflammatory syndrome (Meng et al., 2015), and inflammatory bowel disease (Günther et al., 2011). Notably, Pierdomenico, M. *et al.* reported that both RIP3 and MLKL are highly expressed in children with IBD, who are often accompanied by symptoms of intestinal mucosal inflammation (Pierdomenico et al., 2014). These results above imply that RlP3-dependent necroptosis may functionally involve in the pathogenesis of colitis. Here, we show that curcumin pretreatment remarkably decreased the expression of p-RIP3 and p-MLKL in TNF-α-induced SIRS mice. Interestingly, curcumin does not affect RIP1 protein or phosphorylation levels either *in vivo* or *in vitro*. Similar to ours, Moujalled, D. M. *et al.* stated that in the absence of RIP1, TNF-α can still activate RIP3 and cause necroptosis (Moujalled et al., 2013). Combined with the results reported by Newton, K. *et al.* that “tissue-specific RIP3 deletion identified intestinal epithelial cells as the major target organ” (Newton et al., 2016), it shows that curcumin selectively inhibits necroptosis by targeting RIP3 to achieve the purpose of treating UC.

Additionally, the intestinal mechanical barrier is mainly composed of the mucus layer, intestinal epithelial cells and intercellular TJs, which is the basis for maintaining the structure and function of the intestinal mucosal barrier (Zeisel et al., 2019). IBD is often accompanied by abnormal expression of TJs (Tanaka et al., 2015), so we also investigate curcumin’s involvement in probable mechanisms of intestinal epithelial cell permeability. We speculate that a further mechanism may be to disrupt the epithelial barrier by inhibiting the expression of junctional molecules, promoting the penetration of bacteria and cytotoxic substrates into intestinal mucosa, thereby exacerbating inflammation *in vivo*. Therefore, we analyze the curative effect of curcumin on the expression of TJs in the DSS-induced UC model by immunofluorescence and Western blotting. Here, we find a robust increase of occludin and ZO-1 levels with curcumin treatment in undergoing necroptosis, which confirms the close relationship between RIP3-dependent necroptosis and intestinal epithelial barrier dysfunction (Ibrahim et al., 2020).

In conclusion, our study shows that curcumin has a positive role in colitis-induced intestinal epithelial injury by inhibiting the expression of p-RIP3 and p-MLKL by targeting RIP3, decreasing inflammatory cytokines expression, and inducing intestinal epithelial barrier integrity. We demonstrated that curcumin treatment may be an effective therapeutic approach for the management or collaborative treatment of IBD, including UC in the future.

## Data Availability

The original contributions presented in the study are included in the article/Supplementary Material, further inquiries can be directed to the corresponding authors.
