# Corrigendum: Curcumin alleviates experimental colitis in mice by suppressing necroptosis of intestinal epithelial cells

**DOI:** 10.3389/fphar.2023.1240661

**Published:** 2023-06-28

**Authors:** Yuting Zhong, Ye Tu, Qingshan Ma, Linlin Chen, Wenzhao Zhang, Xin Lu, Shuo Yang, Zhibin Wang, Lichao Zhang

**Affiliations:** ^1^ Department of Pharmacy, Shanghai Municipal Hospital of Traditional Chinese Medicine, Shanghai University of Traditional Chinese Medicine, Shanghai, China; ^2^ Department of Pharmacy, Shanghai East Hospital, School of Medicine, Tongji University, Shanghai, China; ^3^ Department of Critical Care Medicine, School of Anesthesiology, Naval Medical University, Shanghai, China; ^4^ Longhua Hospital Affiliated to Shanghai University of Traditional Chinese Medicine, Shanghai, China

**Keywords:** curcumin, necroptosis, colitis, intestinal epithelial cells, RIP3

In the published article, there was an error in **Affiliations 1 and 2**. Instead of “Department of Pharmacy, Shanghai Municipal Hospital of Traditional Chinese Medicine, Shanghai, China,” affiliation 1 should be “Department of Pharmacy, Shanghai Municipal Hospital of Traditional Chinese Medicine, Shanghai University of Traditional Chinese Medicine, Shanghai, China.” Instead of “Department of Pharmacy, Shanghai East Hospital, Tongji University, Shanghai, China,” affiliation 2 should be “Department of Pharmacy, Shanghai East Hospital, School of Medicine, Tongji University, Shanghai, China.”

The authors apologize for this error and state that this does not change the scientific conclusions of the article in any way. The original article has been updated.

