# Supplementary material for: Curcumin alleviates experimental colitis in mice by suppressing necroptosis of intestinal epithelial cells
Source: Front Pharmacol. 2023 Apr 7;14:1170637. doi: 10.3389/fphar.2023.1170637 (PMC10119427; doi:10.3389/fphar.2023.1170637)
Supplement: Supplementary file 1 [file DataSheet1.DOCX]

1. *Induction of necrosis assay in EOL-1cells*

Each well of a 96-well plate was seeded with 2 x 10^5^ cells. SM-164 hydrochloride (10 nm) and caspase inhibitor z-VAD-fmk (20 μM) were mixed with the corresponding concentrations of curcumin, and half an hour later h-TNFα (2 ng/100 μl) was added for 6 h of co-stimulation (***P<0.001).


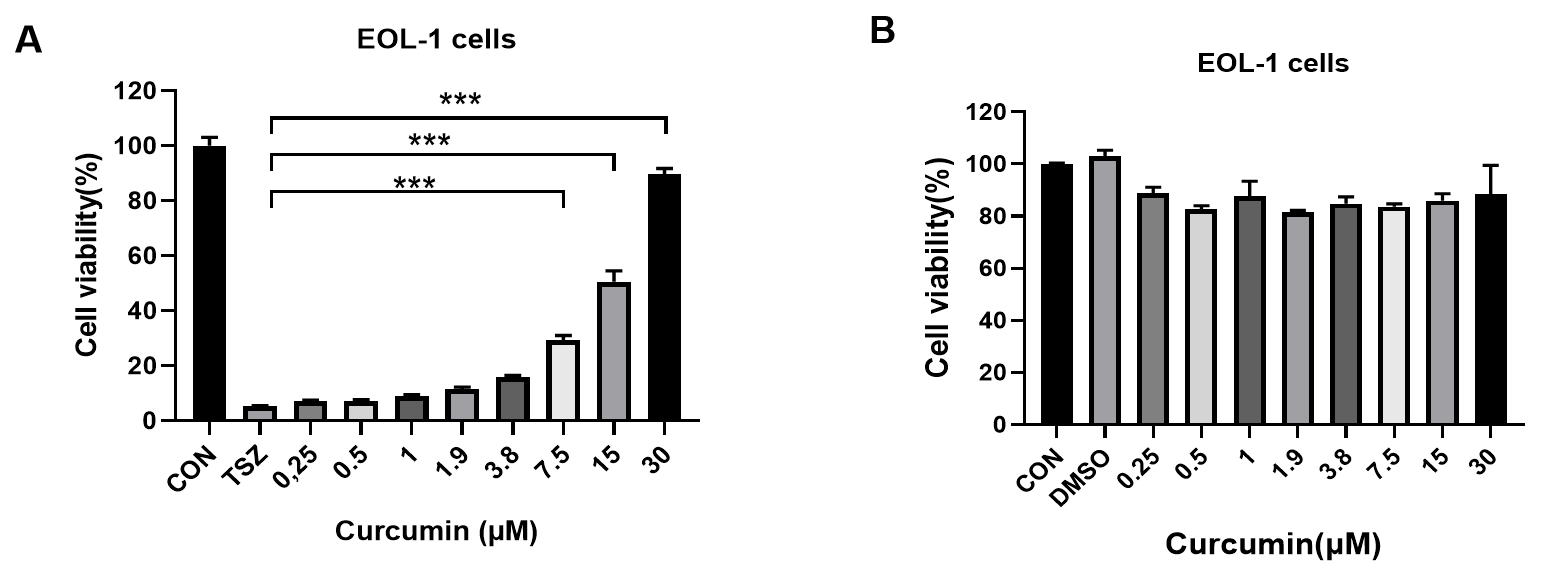


**Supplementary Figure 1.** ***In vitro* anti-necroptosis pharmacological activity of curcumin in EOL-1 cells.** (A) In TSZ (h-TNF-α + SM-164 + Z-VAD-FMK) assay, curcumin protected EOL-1 cells in a dose-dependent manner. (E) Curcumin had no obvious toxic effect on EOL-1 cells within 6 h.

*2. Induction of necrosis assay in L929 cells*

L929 cells were seeded in 96-well plates at 2 x 10^5^ cells per well, and after overnight growth, caspase inhibitor z-VAD-fmk (20 μM) was mixed with the corresponding concentration of curcumin using the medium, and after half an hour h-TNF-α (2 ng/100 μl) was added for co-stimulation for 6 hours.


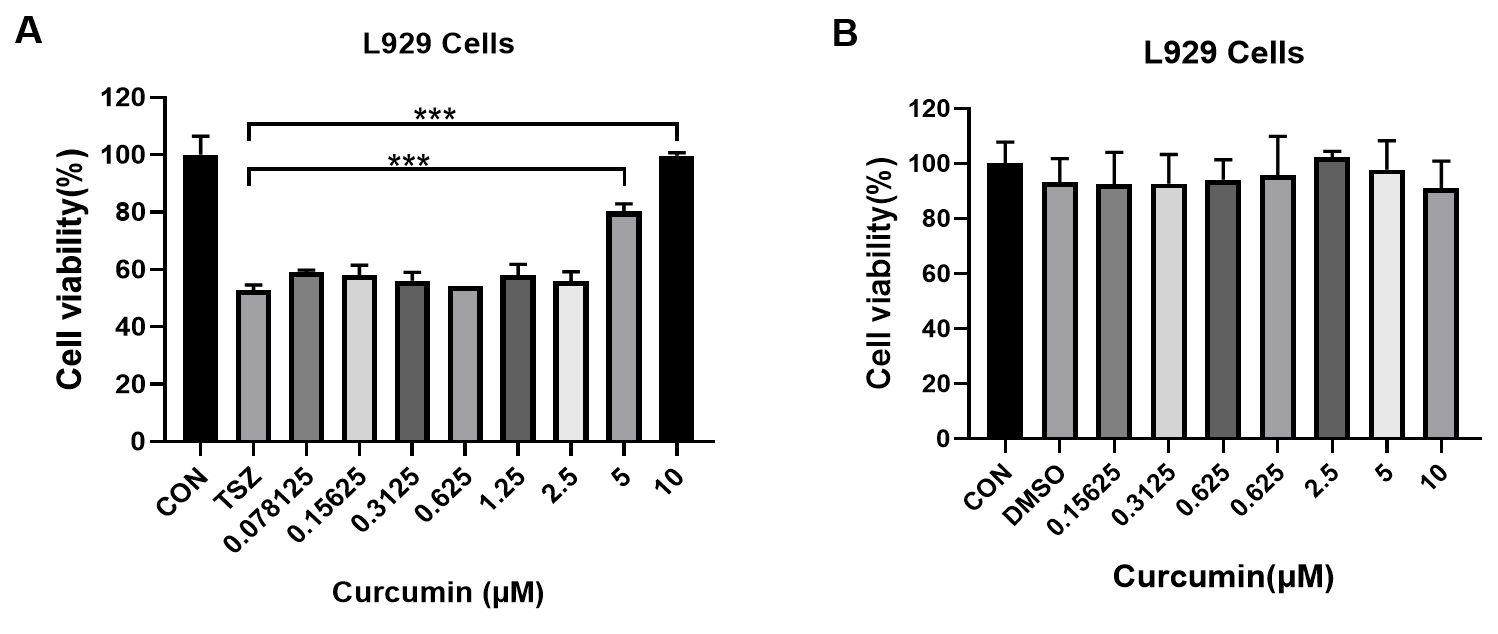


**Supplementary Figure 2.** ***In vitro* anti-necroptosis pharmacological activity of curcumin in L929 cells.** (A) In TZ (h-TNF-α+ Z-VAD-FMK) assay, curcumin protected L929 cells in a dose-dependent manner. (E) Curcumin had no obvious toxic effect on L929 cells within 6 h.

*3. Systemic inflammatory response syndrome (SIRS) in* female C57BL/6 mice

Female C57BL/6 mice (8 weeks, 19-20 g) were randomly divided into Vehicle group (Vehicle), 50mg/kg curcumin group (CUR50), 100mg/kg curcumin group (CUR100) and Necrostatin-1 group (Nec-1), with 7 mice in each group. Z-VAD-fmk was dissolved with the corresponding concentrations of curcumin and Necrostatin-1 using the same solvent. The first dose of z-VAD-fmk and corresponding drugs (180 μg) was intraperitoneally injected 15 min before m-TNF-α (50μg/kg) was injected via the tail vein. The second dose of z-VAD-fmk and the corresponding drugs (70 μg) was given 1 h later, and the initial body temperature was recorded. Body temperature was measured every two hours, and mouse death was recorded.


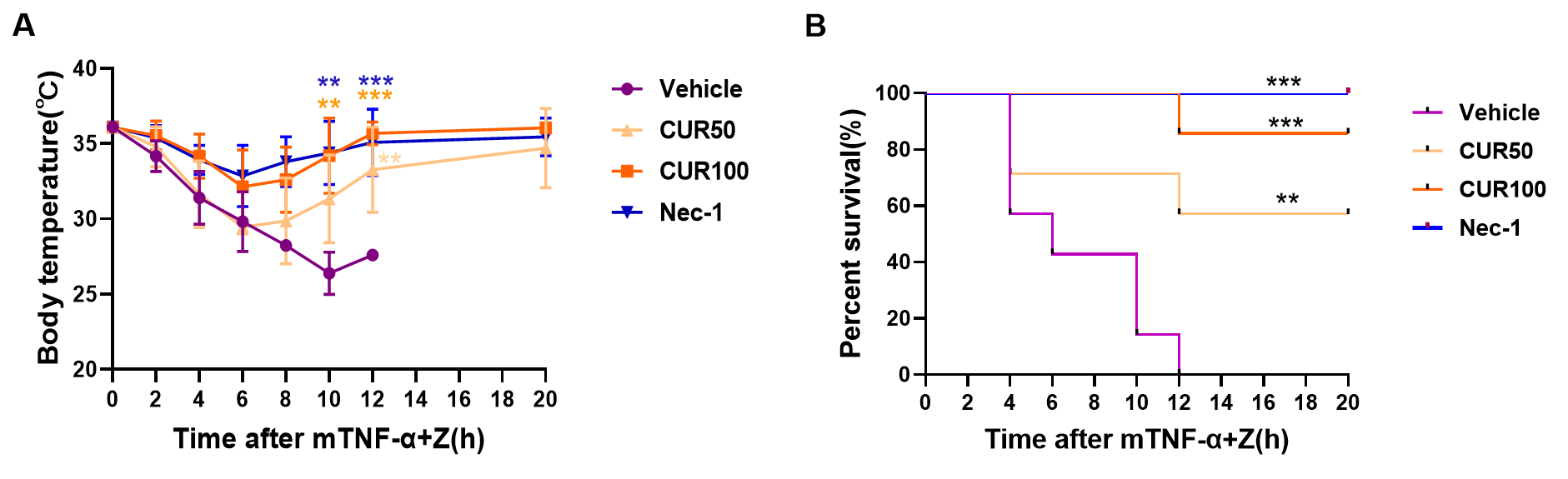


**Supplementary Figure 3.** **Curcumin potently inhibited systemic inflammatory response symptoms in female mice.** (A) Body temperature of female C57BL/6 mice (n=7) injected with m-TNF-α (50 µg/kg) and z-VAD-fmk (250 µg) after treatment with indicated doses of curcumin (100 mg/kg and 50mg/kg) or Necrostatin-1 (5mg/kg) and the survival curves (B). (**P < 0.01; ***P < 0.001 versus Vehicle.)
